# Etude sur le diabète aigu cétosique inaugural dans un hôpital du Centre-Est Tunisien

**DOI:** 10.11604/pamj.2018.31.134.12207

**Published:** 2018-10-23

**Authors:** Ach Taieb, Asma Ben Cheikh, Yosra Hasni, Amel Maaroufi, Maha Kacem, Molka Chaieb, Koussay Ach

**Affiliations:** 1 Service d’Endocrinologie et Diabétologie CHU Farhat Hached, Sousse, Tunisie; 2 Service d’Hygiène Hospitalière CHU Farhat Hached, Sousse, Tunisie

**Keywords:** Diabète de type 1, diabète de type 2, LADA, hyperglycémie, auto-immunité, cétose diabétique, acidocétose diabétique, diabète idiopathique, facteur précipitant, classification du diabète, Type 1 diabetes, type 2 diabetes, LADA, hyperglycemia, autoimmunity, diabetic ketosis, diabetic ketoacidosis, idiopathic diabetes, contributing factor, diabetes classification

## Abstract

La cétose est une complication aiguë du diabète qui consiste en une accumulation de corps cétoniques sanguins. Malgré la haute prévalence du diabète cétosique décrite, il existe très peu d’informations concernant l’épidémiologie de cette complication inaugurale du diabète en Tunisie. L’objectif était de déterminer les caractéristiques épidémiologiques et clinico-biologiques des cétoses inaugurales dans un hôpital du Centre-Est tunisien. Il s’agit d’une étude rétrospective, transversale et exhaustive, à propos de patients admis pour une cétose inaugurale sur une période allant de janvier 2010 à août 2016. La population d’étude a été divisée en 2 groupes selon la présence ou pas d’une auto-immunité anti pancréatique: groupe DAI (diabète de type 1 auto-immun) regroupe tous les patients avec une auto-immunité, et le groupe DNAI (diabète cétosique non auto-immuns) sans auto-immunité. Il s’agit de 391 patients, de sex ratio 266 hommes/125 femmes, d’âge moyen de 34±14,33 ans. La prédominance masculine était nette: 68% dans la population générale. L’âge de la cétose était significativement plus précoce dans le groupe DAI. Un facteur précipitant la cétose était retrouvé chez 77,7% de la population globale d’étude, significativement plus fréquent dans le groupe DAI que dans le groupe DNAI. Le facteur le plus retrouvé était les infections virales. Les Anticorps anti thyroïdiens étaient significativement importants dans le groupe DAI. La cétose est un facteur de décompensation inaugurale fréquent du diabète en Tunisie. La population la plus importante a été décrite chez l’adulte jeune masculin, avec l’absence d’une auto-immunité, et un profil clinique du diabète de type 2.

## Abstract (en)

Ketosis-prone diabetes is an acute complication of diabetes resulting from ketone accumulation in the blood. Despite the high rate of ketosis-prone diabetes described, there is very little information on the epidemiology of this inaugural complication of diabetes in Tunisia. This study aims to determine the epidemiological and clinical features and the laboratory tests parameters of inaugural ketoses in a Hospital in Tunisian. We conducted a retrospective, cross-sectional exhaustive study of patients admitted with inaugural ketosis over the period January 2010 - August 2016. The study population was divided into 2 groups according to the presence or not of anti-pancreatic autoimmunity: the DAI group consisted of all patients with autoimmunity, the DNAI group consisted of all patients without autoimmunity. Our study included 391 patients, with a sex ratio of 226 men/125 women, the average age was 34 ± 14.33 years. There was a male predominance (68%) in the general population. The age of disease onset was significantly lower in the DAI group. A factor that contributed to ketosis onset was found in 77.7% of the overall study population, it was significantly more frequent in the DAI group than in the DNAI group. The most common factor was viral infections. Thyroid antibodies were significantly higher in the DAI group. Ketosis is a common factor leading to inaugural decompensation of diabetes in Tunisia. Young adult male is the most affected group of population reported in the literature, with the absence of autimmunity, and a clinical profile of type 2 diabetes.

## Introduction

La cétose diabétique est une complication fréquente et aiguë du diabète [1]. Cette complication consiste en une accumulation de corps cétoniques dans le sang, associée ou non à une acidose. L'acidocétose diabétique est la conséquence d'une concentration d'insuline trop faible dans le sang, en cas de diabète débutant ou mal compensé par le traitement [2]. Elle est responsable de plus de 100 000 hospitalisations annuelle aux Etats-Unis et de 4 à 9% des dépenses portants sur les patients diabétiques [1]. Bien que la cétose inaugurale se voit le plus souvent dans le diabète de type 1 auto-immun (DAI) chez un sujet jeune [3], plusieurs auteurs ont noté des cas aigus de diabète cétosique non auto-immuns (DNAI) [4-8]. Dans le diabète de type 1, l’Organisation Mondiale de Santé (OMS) [9] et l’American Association of Diabetes (ADA) [10] distinguent deux catégories: la forme auto-immune, nommée diabète de type 1A, incluant le classique diabète du sujet jeune et le diabète auto-immun lent de l’adulte (LADA), et la forme idiopathique, nommée diabète de type 1B, susceptible de décompensations sur un mode céto-acidosique, sans mise en évidence de marqueurs d’auto-immunité. En contraste au diabète de type 1, le diabète de type 2 est dû à une insulinorésistance avec une défaillance relative de l’insulinosécrétion [7, 11]. Certains patients se présentent dans un tableau d’acidocétose diabétique mais qui recouvrent avec le temps une capacité sécrétoire de la cellule Beta pancréatique [12-14]. Malgré la haute prévalence du diabète cétosique décrite dans le monde [1], il existe très peu d’informations concernant l’épidémiologie de cette complication en Tunisie. C’est dans ce cadre que s’inscrit notre travail qui a pour objectif de déterminer les caractéristiques épidémiologiques, cliniques et biologiques des patients diabétiques nouvellement diagnostiqués sous une forme cétosique ou acido-cétosique durant une période de six mois au service d’Endocrinologie au CHU Farhat Hached de Sousse.

## Méthodes

### Type et population à l’étude

Il s’agissait d’une étude rétrospective, transversale et exhaustive, menée dans le service d’Endocrinologie et de Diabétologie au CHU Farhat Hached de Sousse à propos de patients chez qui le diagnostic de diabète a été posé à la suite d’une cétose ou une acidocétose inaugurale sur une période allant de janvier 2010 à août 2016. Ainsi, tout patient ayant les critères de cétose diabétique selon les recommandations de l’American Association of Diabetes (une hyperglycémie > 13.8 mmol/L, une présence de corps cétoniques urinaires et/ou sanguins et un pH < 7,3 pour définir l’acidose) [2] ont été inclus dans l’étude. La population d’étude a été divisée en 2 groupes selon la présence ou pas d’une auto-immunité anti pancréatique attestée par la positivité des anticorps anti pancréatiques: anticorps anti acide glutamique décarboxylase (Ac anti GAD) et/ou anticorps anti-tyrosine phosphatase IA2 (Ac anti IA2). Le groupe DAI regroupe tous les patients avec une auto-immunité pancréatique avérée, et le groupe DNAI les patients sans auto-immunité (Ac anti GAD et Ac anti IA2 négatifs).

### La collecte des données

Les données cliniques familiales et personnelles de la population d’étude ont été recueillies grâce au dossier d’hospitalisation des patients au service. Les facteurs précipitants la cétose ont été étudiés chez tous les patients de la population d’étude. Les données rassemblées portaient sur les éléments suivants: les ATCDS familiaux: diabète de type 1 et type 2, obésité, hypertension artérielle, maladies auto-immunes; les ATCDS personnels métaboliques et de maladies auto-immunes; les différents éléments de l’examen clinique à l’admission: l’examen clinique retenu a été fait au moment de l’hospitalisation en urgence (Il comportait le poids, indice de masse corporelle, le tour de taille, la recherche d’Acanthosis Nigricans, vitiligo et de goitre ainsi que la mesure des chiffres tensionnels); les facteurs précipitant la cétose diabétique à savoir les infections, le stress, le régime diététique, une prise médicamenteuse, une grossesse en cours, l’éthylisme et la situation de jeûne de ramadan; la biologie comportait une glycémie à jeun, une HbA1c, gaz du sang, bilan lipidique, la créatininémie et la recherche d’albuminurie.

Les analyses biologiques de la cétose diabétique associée ou pas avec une acidose ont été obtenus le jour d’hospitalisation du patient. La glycémie était mesurée par méthode enzymatique. L’acétonurie était détectée par bandelettes urinaires. Les anticorps anti GAD et les anticorps anti IA2 ont été mesurés par méthode IRMA (Immuno-radiometric Assay) dans le laboratoire de Physiologie du CHU Farhat Hached Une recherche des maladies auto-immunes associées au moment du diagnostic a été faite grâce au dosage des Ac anti TPO pour la thyroïdite d’Hashimoto, les Ac anti Récepteurs à la TSH pour la maladie de Basedow et les Ac anti Gliadine, anti Endomysium et anti Transglutaminase pour la maladie Cœliaque.

### Analyse des données

Les résultats obtenus ont été étudiés et analysés par le logiciel de statistique SPSS V23.0. Afin de comparer les patients DAI par rapport à ceux ayant un DNAI, nous avons utilisé le test « t » de Student, les variables quantitatives et le test du Chi-deux lorsque les conditions de validité le permettaient pour les variables qualitatives. Le seuil de significativité (p) était fixé à 0,05.

## Résultats

Il s’agissait de 391 patients dont la majorité était de sexe masculin (68%, n = 266) avec un sex ratio de 2,1. L’âge moyen était de 34 ± 14,33 ans et des extrêmes allant de 13 ans à 77 ans. Dans tous les groupes étudiés la prédominance masculine, avec 62,7% (n = 158) dans le groupe DAI et 71,7% (n = 233) dans le groupe DNAI. L’âge de la cétose était significativement plus précoce dans le groupe DAI: 27 ans en moyenne, que dans le groupe DNAI: 38 ans en moyenne (p < 10^-3^). Les antécédents familiaux des différentes populations d’études sont étudiés dans le Tableau 1. Les antécédents familiaux de diabète de type 2 et d’obésité étaient significativement plus fréquents dans le groupe DNAI (p < 10^-3^ et p < 0,033 respectivement). Les antécédents familiaux de diabète de type 1 et d’auto-immunité familiale étaient significativement plus fréquents dans le groupe DAI (p < 10^-3^). L’examen clinique des patients dans les différents groupes est analysé dans le Tableau 2. La durée des signes cardinaux était significativement plus courte dans le groupe DAI (p < 10^-3^). Le groupe DNAI présentait un surpoids et une obésité plus importants que dans le groupe DAI (p < 10^-3^). Les patients avec un IMC > 25 kg/m^2^ ainsi que ceux avec un tour de taille > 94 cm chez les hommes et un tour de taille > 80 cm chez les femmes étaient significativement plus importants dans le groupe DNAI que dans le groupe DAI (p < 10^-3^). Un facteur précipitant la cétose était retrouvé chez 77,7% de la population globale d’étude, significativement plus fréquent dans le groupe DAI que dans le groupe DNAI (p < 10^-3^). Les facteurs les plus retrouvés étaient les infections principalement de type virales qui étaient plus retrouvées dans le groupe DAI que dans le groupe DNAI (p < 0,035). Le stress psychique était plus fréquent que le stress physique et était plus présent dans le groupe DAI que dans le groupe DNAI (p < 0,005). L’excès calorique était aussi fréquemment retrouvé chez 26,8% de la population d’étude. Le résumé des facteurs précipitants la cétose sont regroupés dans le Tableau 3. L’analyse biologique a montré une glycémie moyenne = 17,17 ± 5,66mmol/L, et une HBA1C moyenne = 12,17 ± 2,31%. Il n’y avait pas de différences significatives entre les groupes concernant la glycémie et l’HBA1C. Par contre, le groupe DAI avait une tendance de LDL bas < 1g/L significativement plus importante que dans le groupe DNAI, qui avait une tendance significativement plus importante à l’hyper LDL (p < 10^-3^). Le pH était significativement plus acide dans le groupe DAI que dans le groupe DNAI (p < 10^-3^). L’ensemble des résultats biologiques sont regroupés dans le Tableau 4. Un syndrome métabolique a été retrouvé chez 8,2% de la population totale, tous appartenant au groupe DNAI (p < 10^-3^). L’exploration sérologique auto-immune pratiquée dans le groupe DAI au cours de l’hospitalisation a révélé une prédominance significative des Ac anti GAD présents dans 90,5% des cas par rapport aux Ac anti IA2 présents dans 39.2% des cas seulement (p < 10^-3^). Des Ac anti maladies cœliaques ont été dosés aussi avec une positivité de 2%, significativement plus présents dans le groupe DAI que dans le groupe DNAI: 4,4% contre 1,3% (p < 10^-3^). Une recherche des Ac anti thyroïdiens a montré la présence d’Ac anti TPO et d’Ac anti Récepteurs à la TSH présents dans 3,3% et 3,6% des cas respectivement dans la population générale avec une prédominance significative dans le groupe DAI: 6,3% contre 1,3% dans le groupe DNAI pour les Ac anti Thyroperoxydase (p < 0.006) et 7,6% contre 0,9% respectivement pour les groupes DAI et DNAI (p < 10^-3^) concernant les Ac anti Récepteurs à la TSH.

**Tableau 1:** Caractéristiques épidémiologiques des différents groupes et fréquence des antécédents familiaux

Variables	Tous les patients	DAI	DNAI	p
Nombre de patients	391	158	233	
Age (ans), moyenne ET	34,09±14,33	27,36±25	38,65±14,26	<10^-3^
homme, n(%)	266(68)	99(62,7)	167(71,7)	0.061
Antécédents familiaux de Diabète de type 2, n(%)	192(49,1)	57(36,1)	135(57,9)	<10^-3^
Antécédents familiaux de Diabète de type 1, n(%)	67(17,1)	49(31)	18(7,7)	<10^-3^
Antécédents d'obésité familiale, n(%)	52(13,3)	14(8,9)	38(16,3)	0.033
Hérédité familiale d'HTA, n(%)	167(42,7)	61(38,6)	106(45,5)	0.177
Hérédité familiale d'auto-immunité, n(%)	106(27,1)	61(38,6)	45(19,3)	<10^-3^

ET: Ecart type

**Tableau 2:** Examen clinique à l’admission des différents groupes d’étude

Variables	Tous les patients	DAI	DNAI	p
Durée des signes cardinaux (semaines), moyenne ET	8,86±11,25	5,34±5,4	11,25±13,38	<10^-3^
Amaigrissement (Kg), moyenne ET	8,95±4,12	8,56±3,8	9,22±4,3	0.108
IMC (25 et 30 kg/m²), n(%)	136(34,8)	30(19)	106(45,5)	<10^-3^
IMC >30 kg/m², n(%)	38(9,7)	4(2,5)	34(14,6)	<10^-3^
Tour de taille > 80 cm chez les femmes, n(%)	71(56,8)	23(39)	48(72,7)	<10^-3^
Tour de taille > 94 cm chez les hommes, n(%)	81(30,5)	6(6,1)	75(44,9)	<10^-3^
HTA personnelle, n(%)	36(9,2)	5(3,2)	31(13,3)	0.001
maladie auto-immune connue, n(%)	18(4,6)	13(8,2)	5(2,1)	0.005
Tension artérielle systolique (mmHg), moyenne ET	11,79±1,3	11,35±1,11	12,09±1,34	<10^-3^
Tension artérielle Diastolique (mmHg), moyenne ET	7,24±0,94	6,91±0,88	7,46±0,92	<10^-3^
Acanthosis Nigricans, n(%)	46(11,8)	2(1,3)	44(18,9)	<10^-3^
Vitiligo, n(%)	7(1,8)	4(2,5)	3(1,3)	0.447
Goitre, n(%)	12(3,1)	8(5,1)	4(1,7)	0.113
Complication dégénérative au moment du diagnostic, n(%)	19(4,9)	0(0)	19(8,2)	<10^-3^

ET: Ecart type; IMC: Indice de Masse Corporelle

**Tableau 3:** Etude des différents facteurs précipitants la cétose diabétique dans les différents groupes

Variables		Tous les patients n (%)	DAI n (%)	DNAI n (%)	p
Facteurs précipitants n(%)		304(77,7)	146(92,4)	158(67,8)	<10^-3^
Infections n(%)	Virales	81(20,7)	41(25,9)	40(17,2)	0.035
	Bactériennes	6(16,4)	21(13,3)	43(18,5)	0.176
Stress n(%)	Physique	21(5,4)	8(5,1)	13(5,6)	0.824
	Psychique	141(36,1)	70(44,3)	71(30,5)	0.005
Troubles alimentairesn(%)	Boissons hypertoniques	78(19,9)	36(22,8)	42(18)	0.248
	régime hypercalorique	27(6,9)	14(8,9)	13(5,6)	0.208
Alcool n(%)	47(12)	22(13,9)	25(10,7)	0.341	
Jeûne de Ramadan n(%)	14(3,6)	8(5,1)	6(2,6)	0.194	
Corticothérapie n(%)	10(2,6)	0(0)	10(4,3)	0.021	
Grossesse n(%)	9(2,3)	6(3,8)	3(1,3)	0.2	
Hypokaliémie n(%)	11(2,8)	7(4,4)	4(1,7)	0.2	

ET: Ecart type

**Tableau 4:** Biologie comparatives entre les différents groupes

Variables	Tous les patients	DAI	DNAI	p
Glycémie à jeun (mmol/L), moyenne ET	17,17± 5,66	16,52±5,44	17,61±5,77	0.062
HBA1C (%), moyenne ET	12,17±2,31	12,11±2,26	12,22±2,34	0.669
LDL (g/L), moyenne ET	1,09±0,47	0,87±0,31	1,23±0,5	<10^-3^
LDL > 1 g/L n(%)	182(46,5)	34(21,5)	148(63,5)	<10^-3^
HDL (mmol/L), moyenne ET	1,24±0,46	1,36±0,45	1,16±0,45	<10^-3^
Albuminurie (mg/L), moyenne ET	14,03±8,68	12,23±6,99	15,26±9,48	<10^-3^
pH, moyenne ET	7,34±0,10	7,28±0,13	7,38±0,05	<10^-3^
Créatinine (µmol/L), moyenne ET	62,26±18,53	58,17±15,98	64,65±19,76	0.001

HBA1C: Hémoglobine glyquée

## Discussion

Dans cette étude, nous avons cherché à caractériser une population se présentant pour une cétose inaugurale. La cétose diabétique représente un problème de santé dans le monde [15]. Dans des séries africaines, la prévalence hospitalière de l’acidocétose diabétique chez l’adulte était de 5 à 6,6% [16]. L’étude d’incidence a été fortement analysée dans la population jeune [3, 17]. Il est bien connu que l’acidocétose se présente le plus souvent chez les enfants et adolescents diabétiques de type 1 [18], ce qui rejoint notre étude qui a retrouvé une corrélation positive entre la survenue de la cétose et l’âge jeune chez les patients porteurs d’Ac anti GAD et/ou Ac anti IA2 positifs. Dans une étude menée chez les diabétiques de type 1 nouvellement diagnostiqués, l’incidence de l’acidocétose étudiée sur 2 années était de 426 enfants [3]. Le sex ratio de notre population d’étude montrait une nette prédominance masculine significative, comme l’a décrite plusieurs équipes chinoises et africaines [15, 19]. Les facteurs précipitants les plus retrouvés dans notre travail étaient l’infection virale et le stress physique et psychiques. La perte des cellules Bêta pancréatiques par apoptose suite aux viroses a été étudiée par Beeck *et al.* [20] qui ont noté que les virus les plus incriminés étaient les entérovirus, dont le Coxsackievirus. La survenue de maladie auto-immune dont fait partie le diabète de type 1 incrimine le facteur stress comme l’illustre de nombreux travaux, mais aussi des facteurs génétiques incluant le HLA [21, 22]. Le diabète de type 1 touche les enfants chez qui on retrouve le stress scolaire et familial, contrastant au stress professionnel chez l’adulte, mais aussi la détention carcérale. Les autres facteurs précipitant sans à ne pas omettre et peuvent inclure la prise médicamenteuse, la grossesse mais aussi la période de jeûne de ramadan.

La population de diabétique de type 1 de notre étude avait une association significative avec l’auto-immunité familiale et était associée à une plus forte prévalence de dysthyroïdies auto-immunes. Dans le travail de Korner et al, les auteurs ont prouvé que les dysthyroïdies auto-immunes étaient la pathologie la plus fréquente avec la maladie cœliaque, ce qui rend important le dépistage de ces pathologies à la découverte du diabète. Le diabète de type 1 survient le plus souvent chez le sujet maigre, sans antécédents de maladies métaboliques, comme nous l’avions retrouvé dans notre étude. Les diabétiques chez qui la sérologie était négative mais possédant un morphotype typique du diabète auto-immun du sujet jeune peuvent être un authentique diabète de type 1, mais dû à des anticorps qui ne sont pas dosés dans notre laboratoire à savoir les anticorps anti ICA et anti ZnT8. En contraste avec le classique morphotype du sujet jeune et maigre, il existe un tout autre aspect de diabète du sujet jeune avec des anticorps négatifs avec un morphotype ressemblant à un diabète de type 2, ce qui a incité certains auteurs à le décrire comme un diabète de type 1 b ou diabète idiopathique [8]. Le diabète de type 1b ayant une présentation initiale aiguë qui se caractérise par une hyperglycémie sévère et une cétose comme au cours du diabète de type 1A. Après l’initiation de l’insulinothérapie, une rémission prolongée est souvent possible avec un contrôle glycémique satisfaisant malgré l’arrêt de l’insuline [12, 23]. La détection de ce type de diabète nécessite le dosage du peptide C pour affirmer le rétablissement de la sécrétion pancréatique, chose qui n’a pas été faite dans notre étude. Des niveaux variables d’insulinorésistance ont été observés, en particulier chez les sujets obèses [24, 25]. Les mécanismes moléculaires à l’origine de l’altération transitoire de la sécrétion d’insuline sont encore inconnus, mais pourraient impliquer des mécanismes de glucotoxicité, une altération de la sécrétion du glucagon, l’effet du stress et des facteurs génétiques [26]. Ce type de diabète est plus fréquent dans la zone de l’Afrique du nord et américaine [16]. Récemment, une association entre le diabète de l’Africain et l’infection HHV-8 a été retrouvée, laissant suggérer que la phase aigüe de décompensation cétosique pourrait être liée à cette infection virale [27]. Ce type de diabète serait encore plus intéressant à découvrir puisqu’il implique la reprise de la sécrétion des cellules Bêta pancréatique, et pourrait donc exposer à des problèmes de surexposition à l’insuline à type d’épisodes d’hypoglycémie ou de prise de poids [28].

L’autre forme de diabète de type 1 survenant chez le sujet âgé, est le LADA, qui est une forme frontière avec le diabète de type 2 [29]. Certains auteurs évoqueraient le rôle de l’inflammation dans la genèse de l’auto-immunité pancréatique [30]. Brooks-Worrell *et al.* ont discuté dans leur travail de la continuité possible entre les différents types de diabète, en décrivant le chevauchement de leurs cliniques jusqu’à envisager les diabètes comme un spectre continu. Certains auteurs proposent la nomenclature du diabète selon la classification (A/β): a étant pour la présence de l’auto-immunité et β pour la positivité de la fonction des cellules ß pancréatique. Cette classification A/B a montré une haute précision et s’est révélée être un facteur de meilleur contrôle glycémique et de gestion dans la thérapeutique envisagée dans ces types de diabètes. La cétose peut survenir à un âge avancé, et être un mode de révélation du diabète de type 2. Le groupe non auto-immun de notre population était fortement corrélé aux antécédents de diabète de type 2 allant jusqu’à retrouver d’authentiques syndromes métaboliques exclusifs à ce groupe. Il est logique dès lors de ne plus retrouver l’association aux maladies auto-immunes retrouvées dans le groupe de diabète de type 1, mais plutôt une corrélation positive à l’obésité et à une présence plus importante d’Acanthosis Nigricans.

Newton *et al.* [18] ont décrit l’acidocétose diabétique chez le sujet sans auto-immunité le plus souvent chez les africains et les afro-américains [p, 001]. La présence de la cétose chez les diabétiques de type 2 à un taux élevé par rapport aux diabétiques de type 1 peut être expliqué par 2 faits : le premier étant que le travail mené portait sur une population de sujet hospitalisé dans un service d’endocrinologie adulte, d’où le pourcentage élevé de sujet d’âge avancé corrélé avec la fréquence du diabète de type 2 dans cette tranche d’âge. Le deuxième fait, étant que le diabète de type 1 est fortement présent dans la population caucasienne alors que ce travail a été menée sur une population exclusivement Nord-Africaine. Ce groupe présente un large spectre de patients assez hétérogène, puisque les extrêmes d’âges varient de l’âge jeune à un âge avancé, avec une possibilité de reprise de l’insulinosécrétion après une durée ultérieure. La durée des signes cardinaux dans le diabète de type 2 est rarement aigue. Dans notre étude, les signes cardinaux pouvaient être précoces chez certains individus sans auto-immunité. Ce type de diabète de type 2 aigu cétosique atypique a été décrit par plusieurs équipes, qui ont même retrouvé la prédominance masculine dans cette population, comme attestée par notre travail. Une revue montre que l’acidocétose pouvait se rencontrer chez des sujets diabétiques avec un morphotype mimant un diabète de type 2 atypique notamment par l’absence de la prévalence de l’auto-immunité et l’obésité. Le dosage de l’insuline pancréatique et du peptide C pourraient être un marqueur de prédiction du retour de l’insulinosécrétion physiologique et permettraient d’évaluer l’arrêt de l’insuline au long cours. L’augmentation de l’incidence du diabète de type 2 cétosique rencontrée le plus souvent chez les sujets de sexe masculin relèverait d’autres facteurs et mécanismes tels que l’influence hormonale, la répartition de la masse grasse, et la modification de l’insulino sensibilité [15]. Les facteurs environnementaux comme la prise de boissons hypertoniques, l’écart de régime et l’activité physique couplés à des facteurs génétiques en cours de recherche sont fortement incriminés, et leurs interactions contribuent à la genèse de ce syndrome [25]. Ce travail présente plusieurs limites comme nous l’avions évoqué. La première étant le fait que ce soit une étude rétrospective, basée sur l’étude de dossiers des patients. La deuxième concernait la population d’étude qui était quasi exclusivement faite de sujets adultes, ce qui a pu rendre la prévalence de la cétose du diabète de type 1 moindre chez les jeunes par rapport à la population adulte. Ensuite, la non disponibilité du dosage des auto-anticorps autres que les Ac anti GAD et les anti IA2, peut être un biais dans le typage du diabète chez le diabétique avec un authentique profil de diabète de type 1. Concernant le profil évolutif, l’absence de dosage de l’insuline et du peptide C constitue une limite pour l’évaluation de la fonction de la cellule Beta pancréatique au cours du suivi de ces diabètes atypiques.

## Conclusion

Cette étude illustre la nette présence de la cétose comme étant un facteur de révélation inaugurale du diabète en Tunisie. Bien que ce travail ait été dirigé dans un service d’endocrinologie adulte, une proportion assez élevée a été décrite chez des adolescents et des sujets jeunes, ce qui rend cette décompensation plus spécifique dans le jeune âge. Cette étude a montré que la population la plus importante a été décrite chez l’adulte jeune et de moyen âge, avec l’absence d’une auto-immunité, et un profil clinique du diabète de type 2. La prédominance masculine a été retrouvée chez les 2 groupes d’étude conformément à la littérature. Les facteurs à l’origine de la cétose étudiés confirment la prédominance de l’infection et l’association aux facteurs de stress et de diététique hypertonique. La difficulté dans la classification de certains patients porteurs d’atypies cliniques et sérologiques incite à reconsidérer les moyens de classifications usuelles en faveur d’autres moyens telle que la classification A/B. Cette classification ne peut se faire que par l’évaluation de la fonction Beta pancréatique, qui permet d’aider à la gestion du diabète.

### Etat des connaissances actuelles sur le sujet

- La cétose diabétique est une complication fréquente et aiguë du diabète. Elle est responsable de plus de 100 000 hospitalisations annuelle aux Etats-Unis et de 4 à 9% des dépenses portants sur les patients diabétiques;
- Bien que la cétose inaugurale se voit le plus souvent dans le diabète de type 1 auto-immun chez un sujet jeune, plusieurs auteurs ont noté des cas aigus de diabète cétosique non auto-immuns;
- Dans le type 1 il existe deux catégories: La forme auto-immune, nommée diabète de type 1A, incluant le classique diabète du sujet jeune et le diabète auto-immun lent de l’adulte (LADA), et la forme idiopathique, nommée diabète de type 1B, susceptible de décompensations sur un mode céto-acidosique, sans mise en évidence de marqueurs d’auto-immunité, et qui recouvre leur insulino sécrétion avec le temps.

### Contribution de notre étude à la connaissance

- Malgré la haute prévalence du diabète cétosique décrite dans le monde, il existe très peu d’informations concernant l’épidémiologie de cette complication en Tunisie et en Afrique ; les facteurs de précipitation de la cétose diabétique bien décrits sont aussi peu cités dans la littérature;
- Cette étude démontre la difficulté de la caractérisation du diabète selon le schéma classique: type 1 et type 2; vu la disparité des profils cliniques qui rendent un potentiel chevauchement dans la clinique du diabète auto-immun avec le diabète de type 2;
- Cette étude a porté sur une population d’étude, avec des résultats qui rejoignent les résultats de la bibliographie internationale en termes de significativité; elle initie un ensemble d’autres travaux qui seront dirigés en vue d’utiliser de nouvelles méthodes de classification du diabète.

## Conflits d’intérêts

Les auteurs ne déclarent aucun conflit d'intérêts.

## Contributions des auteurs

Tous les auteurs ont contribué à la conduite de ce travail. Tous les auteurs déclarent également avoir lu et approuvé la version finale du manuscrit.
